# Supplementary material for: New-Onset Rheumatic Immune-Mediated Inflammatory Diseases Following SARS-CoV-2 Vaccinations until May 2023: A Systematic Review
Source: Vaccines (Basel). 2023 Oct 8;11(10):1571. doi: 10.3390/vaccines11101571 (PMC10610967; doi:10.3390/vaccines11101571)
Supplement: Supplementary file 1 [file vaccines-11-01571-s001.zip › Supplementary file S2. Search strategies.pdf]

*Systematic review: new rheumatic immune-mediated inflammatory diseases (R-IMIDs) after SARS CoV-2 vaccinations*

*30 May 20*

## Search strategies

### A.1 Medline

Ovid MEDLINE(R) ALL <1946 to May 24, 2023> Results per line

Date: 25/05/2023

| | | |
|---|---|---|
| 1 | exp Arthritis, Rheumatoid/ | 125199 |
| 2 | exp Axial Spondyloarthritis/ | 16644 |
| 3 | Spondylitis, Ankylosing/ | 16422 |
| 4 | Arthritis, Reactive/ | 4060 |
| 5 | Arthritis, Psoriatic/ | 8022 |
| 6 | exp Crystal Arthropathies/ | 15744 |
| 7 | exp Gout/ | 13865 |
| 8 | exp Chondrocalcinosis/ | 2235 |
| 9 | exp Connective Tissue Diseases/ | 346656 |
| 10 | Antiphospholipid Syndrome/ | 9262 |
| 11 | exp Lupus Erythematosus, Systemic/ | 66856 |
| 12 | Sjogren's Syndrome/ | 14563 |
| 13 | exp Scleroderma, Systemic/ | 23012 |
| 14 | exp Myositis/ | 22425 |
| 15 | Dermatomyositis/ | 9030 |
| 16 | Polymyositis/ | 2723 |
| 17 | Still's Disease, Adult-Onset/ | 1683 |
| 18 | exp Anti-Neutrophil Cytoplasmic Antibody-Associated Vasculitis/ | 12278 |
| 19 | Granulomatosis with Polyangiitis/ | 7812 |
| 20 | exp Microscopic Polyangiitis/ | 733 |
| 21 | Polyarteritis Nodosa/ | 6230 |
| 22 | Takayasu Arteritis/ | 4594 |
| 23 | Giant Cell Arteritis/ | 7470 |

24 Polymyalgia Rheumatica/ 2792

25 Polychondritis, Relapsing/ 1526

26 exp Sarcoidosis/ 26956

27 Behcet Syndrome/ 10054

28 IgA Vasculitis/ 4935

29 Cryoglobulinemia/ 3330

30 Cogan Syndrome/ 258

31 Immunoglobulin G4-Related Disease/ 1190

32 (rheumatic immune mediated inflammatory diseases or R-IMID$ or Inflammatory arthritis or rheumatoid arthritis or Axial Spondyloarthritis or ankylosing spondylitis or reactive arthritis or psoriatic arthritis or crystal arthritis or gout or pseudogout or chondrocalcinosis or "remitting seronegative symmetrical synovitis with pitting oedema" or "remitting seronegative symmetrical synovitis with pitting edema" or connective tissue disease$ or CTD or anti-phospholipid syndrome or (systemic lupus erythematosus or SLE) or lupus or (systemic sclerosis or scleroderma) or inflammatory myopathy or myositis or dermatomyositis or polymyositis or necrotizing autoimmune myopathy or anti-synthetase syndrome or adult-onset stills disease or Anti-Neutrophil Cytoplasmic Antibody-Associated Vasculitis or ANCA associated vasculitis or "Granulomatosis with polyangiitis" or "Eosinophilic granulomatosis with polyangiitis" or microscopic polyangiitis or Polyarteritis Nodosa or large vessel vasculitis or Takayasu$ arteritis or Giant Cell Arteritis or medium vessel vasculitis or small vessel vasculitis or polymyalgia rheumatica or relapsing polychondritis or sarcoidosis or bechet$ disease or behcet$ syndrome or IgA Vasculitis or Henoch-Schoenlein purpura or Cryoglobulinemia$ or cogan$ syndrome or Immunoglobulin G4-Related Disease or ''IgG4 disease'').mp. 414924

33 or/1-32 571738

34 exp COVID-19 Vaccines/ 21269

35 (chadox1 ncov-19 or ad26covs1 or 2019-ncov vaccine mrna-1273 or bnt162 vaccine).mp. 4809

36 (SARS CoV-2 vaccin$ or Pfizer-Bio-N-tech or Pfizer or Moderna or AstraZeneca or Covishield or Covaxin or Sputnik V or Sinovac or Sinopharm or Corona Vac or Corona virus disease-19 vaccine).mp. 14729

37 ((coronavirus$ or 2019nCoV$ or 19nCoV$ or "2019 novel$" or Ncov$ or "n-cov" or "SARS-CoV-2$" or "SARSCoV-2$" or SARSCoV2$ or "SARS-CoV2$" or "severe acute respiratory syndrome$" or COVID$2) adj3 vaccin$).mp. 36392

38 or/34-37 42535

39 33 and 38 1101

40 (new-onset or new presentation or newly presenting or newly presented or new diagnosis or newly diagnosed or newly developed or "linked with" or "linked to" or associated or post or following or after or "connected to" or "connected with").mp. 11300844

41 39 and 40 870

| | | |
|---|---|---|
| 42 | exp Animals/ | 26382900 |
| 43 | Humans/ | 21259566 |
| 44 | 42 not 43 | 5123334 |
| 45 | 41 not 44 | 869 |

**A.2 Embase**

Embase <1974 to 2023 May 24>Results per line

Date: 25/05/2023

| | | |
|---|---|---|
| 1 | exp rheumatoid arthritis/ | 237322 |
| 2 | exp axial spondyloarthritis/ | 32894 |
| 3 | ankylosing spondylitis/ | 31670 |
| 4 | reactive arthritis/ | 4034 |
| 5 | psoriatic arthritis/ | 30756 |
| 6 | exp crystal arthropathy/ | 26613 |
| 7 | gout/ | 25732 |
| 8 | pseudogout/ | 1267 |
| 9 | exp connective tissue disease/ | 531232 |
| 10 | exp antiphospholipid syndrome/ | 20529 |
| 11 | exp systemic lupus erythematosus/ | 110160 |
| 12 | lupus vulgaris/ | 3387 |
| 13 | Sjoegren syndrome/ | 27760 |
| 14 | exp systemic sclerosis/ | 36983 |
| 15 | exp scleroderma/ | 54045 |
| 16 | exp myositis/ | 47381 |
| 17 | exp dermatomyositis/ | 19335 |
| 18 | polymyositis/ | 9699 |
| 19 | adult onset Still disease/ | 2985 |
| 20 | exp ANCA associated vasculitis/ | 27412 |
| 21 | Wegener granulomatosis/ | 15078 |
| 22 | microscopic polyangiitis/ | 4132 |
| 23 | polyarteritis nodosa/ | 7175 |
| 24 | aortic arch syndrome/ | 3515 |
| 25 | exp giant cell arteritis/ | 13587 |
| 26 | rheumatic polymyalgia/ | 6715 |
| 27 | relapsing polychondritis/ | 2385 |

28 exp sarcoidosis/ 46457

29 Behcet disease/ 17454

30 anaphylactoid purpura/ 7486

31 cryoglobulinemia/ 7289

32 Cogan syndrome/ 843

33 immunoglobulin G4 related disease/ 5099

34 (rheumatic immune mediated inflammatory diseases or R-IMID$ or Inflammatory arthritis or rheumatoid arthritis or Axial Spondyloarthritis or ankylosing spondylitis or reactive arthritis or psoriatic arthritis or crystal arthritis or gout or pseudogout or chondrocalcinosis or "remitting seronegative symmetrical synovitis with pitting oedema" or "remitting seronegative symmetrical synovitis with pitting edema" or connective tissue disease$ or CTD or anti-phospholipid syndrome or (systemic lupus erythematosus or SLE) or lupus or (systemic sclerosis or scleroderma) or inflammatory myopathy or myositis or dermatomyositis or polymyositis or necrotizing autoimmune myopathy or anti-synthetase syndrome or adult-onset stills disease or Anti-Neutrophil Cytoplasmic Antibody-Associated Vasculitis or ANCA associated vasculitis or "Granulomatosis with polyangiitis" or wegener Granulomatosis or "Eosinophilic granulomatosis with polyangiitis" or microscopic polyangiitis or Polyarteritis Nodosa or large vessel vasculitis or Takayasu$ arteritis or aortic arch syndrome or Giant Cell Arteritis or medium vessel vasculitis or small vessel vasculitis or polymyalgia rheumatica or relapsing polychondritis or sarcoidosis or bechet$ disease or behcet$ syndrome or IgA Vasculitis or anaphylactoid purpura or Henoch-Schoenlein purpura or Cryoglobulinemia$ or cogan$ syndrome or immunoglobulin G4 related disease or ''IgG4 disease'').mp. 691340

35 or/1-34 786726

36 exp SARS-CoV-2 vaccine/ 36598

37 (chadox1 ncov-19 or ad26covs1 or 2019-ncov vaccine mrna-1273 or bnt162 vaccine).mp. 1814

38 (SARS CoV-2 vaccin$ or Pfizer-Bio-N-tech or Pfizer or Moderna or AstraZeneca or Covishield or Covaxin or Sputnik V or Sinovac or Sinopharm or Corona Vac or ''Corona virus disease-19 vaccine'').mp. 102617

39 ((coronavirus$ or 2019nCoV$ or 19nCoV$ or "2019 novel$" or Ncov$ or "n-cov" or "SARS-CoV-2$" or "SARSCoV-2$" or SARSCoV2$ or "SARS-CoV2$" or "severe acute respiratory syndrome$" or COVID$2) adj3 vaccin$).mp. 49387

40 or/36-39 119683

41 35 and 40 4194

42 (new-onset or new presentation or newly presenting or newly presented or new diagnosis or newly diagnosed or newly developed or "linked with" or "linked to" or associated or post or following or after or "connected to" or "connected with").mp. 15220564

43 41 and 42 2610

44 limit 43 to human 2493

| | | |
|---|---|---|
| 45 | exp conference paper/ | 2709759 |
| 46 | conference.pt. | 5548357 |
| 47 | or/45-46 | 5570726 |
| 48 | 44 not 47 | 1821 |

### A.3 Cochrane

Cochrane Central Register of Controlled Trials (CENTRAL) and Cochrane Database of Systematic Reviews (CDSR) Results per line

Date: 30/05/2023

#1 MeSH descriptor: [Arthritis, Rheumatoid] explode all trees 7283

#2 MeSH descriptor: [Axial Spondyloarthritis] explode all trees 847

#3 MeSH descriptor: [Spondylitis, Ankylosing] this term only 823

#4 MeSH descriptor: [Arthritis, Reactive] this term only 46

#5 MeSH descriptor: [Arthritis, Psoriatic] this term only 633

#6 MeSH descriptor: [Crystal Arthropathies] explode all trees 682

#7 MeSH descriptor: [Gout] explode all trees 656

#8 MeSH descriptor: [Chondrocalcinosis] explode all trees 26

#9 MeSH descriptor: [Connective Tissue Diseases] explode all trees 12087

#10 MeSH descriptor: [Antiphospholipid Syndrome] this term only 138

#11 MeSH descriptor: [Lupus Erythematosus, Systemic] explode all trees 1420

#12 MeSH descriptor: [Sjogren's Syndrome] this term only 362

#13 MeSH descriptor: [Scleroderma, Systemic] explode all trees 716

#14 MeSH descriptor: [Myositis] explode all trees 316

#15 MeSH descriptor: [Dermatomyositis] this term only 138

#16 MeSH descriptor: [Polymyositis] this term only 60

#17 MeSH descriptor: [Still's Disease, Adult-Onset] this term only 12

#18 MeSH descriptor: [Anti-Neutrophil Cytoplasmic Antibody-Associated Vasculitis] explode all trees 232

#19 MeSH descriptor: [Granulomatosis with Polyangiitis] this term only 107

#20 MeSH descriptor: [Microscopic Polyangiitis] explode all trees 63

#21 MeSH descriptor: [Polyarteritis Nodosa] this term only 40

#22 MeSH descriptor: [Takayasu Arteritis] this term only 43

#23 MeSH descriptor: [Giant Cell Arteritis] this term only 140

#24 MeSH descriptor: [Polymyalgia Rheumatica] this term only 105

#25 MeSH descriptor: [Polychondritis, Relapsing] this term only 1

#26 MeSH descriptor: [Sarcoidosis] explode all trees 306

#27 MeSH descriptor: [Behcet Syndrome] this term only 150

#28 MeSH descriptor: [IgA Vasculitis] this term only 46

#29 MeSH descriptor: [Cryoglobulinemia] this term only 41

#30 MeSH descriptor: [Cogan Syndrome] this term only 8

#31 MeSH descriptor: [Immunoglobulin G4-Related Disease] this term only 13

#32 “rheumatic immune mediated inflammatory diseases” or R-IMID or R-IMIDs or “Inflammatory arthritis” or “rheumatoid arthritis” or “Axial Spondyloarthritis” or “ankylosing spondylitis” or “reactive arthritis” or “psoriatic arthritis” or “crystal arthritis” or gout or pseudogout or chondrocalcinosis or "remitting seronegative symmetrical synovitis with pitting oedema" or "remitting seronegative symmetrical synovitis with pitting edema" or “connective tissue” NEXT disease* or CTD 26811

#33 "anti-phospholipid syndrome" or "systemic lupus erythematosus" or SLE or lupus or "systemic sclerosis" or scleroderma or "inflammatory myopathy" or myositis or dermatomyositis or polymyositis or "necrotizing autoimmune myopathy" or "anti-synthetase syndrome" or "adult-onset stills disease" or "Anti-Neutrophil Cytoplasmic Antibody Associated Vasculitis" or "ANCA associated vasculitis" 7731

#34 "Granulomatosis with polyangiitis" or "Eosinophilic granulomatosis with polyangiitis" or "microscopic polyangiitis" or "Polyarteritis Nodosa" or "large vessel vasculitis" or Takayasu* NEXT arteritis or "Giant Cell Arteritis" or "medium vessel vasculitis" or "small vessel vasculitis" or "polymyalgia rheumatica" or "relapsing polychondritis" or sarcoidosis or Bechet* NEXT disease or Behcet* NEXT syndrome or "IgA Vasculitis" or "Henoch-Schoenlein purpura" or Cryoglobulinemia* or cogan NEXT syndrome or "Immunoglobulin G4-Related Disease" or "IgG4 disease" 2167

#35 {or #1-#34} 38485

#36 MeSH descriptor: [COVID-19 Vaccines] explode all trees 409

#37 "chadox1 ncov-19" or ad26covs1 or "2019-ncov vaccine mrna-1273" or "bnt162 vaccine" 90

#38 "SARS CoV-2" NEXT vaccin* or Pfizer-Bio-N-tech or Pfizer or Moderna or AstraZeneca or Covishield or Covaxin or Sputnik V or Sinovac or Sinopharm or "Corona Vac" or "Corona virus disease-19 vaccine" 7733

#39 (coronavirus* or 2019nCoV* or 19nCoV* or Ncov* or n-cov or SARS-CoV-2 or SARSCoV-2 or SARSCoV2 or SARS-CoV2 or "severe acute respiratory" NEXT syndrome* or COVID) NEAR/3 vaccin* 1808

#40 {or #36-#39} 9169

#41 #35 and #40 456

#42 new-onset or new presentation or newly presenting or newly presented or new diagnosis or newly diagnosed or newly developed or "linked with" or "linked to" or associated or post or following or after or "connected to" or "connected with" 1137116

#43 #41 and #42 in Cochrane Reviews, Cochrane Protocols and Trials 384

## A.4 LitCovid

LitCovid Results

Date: 30/05/2023

("connective tissue disease" OR rheumatic OR arthritis) AND (new-onset OR new presentation OR newly presenting OR newly presented OR new diagnosis OR newly diagnosed OR newly developed OR "linked with" OR "linked to" OR associated OR post OR following OR after OR "connected to" OR "connected with") AND (vaccine OR vaccination)

~510

## A.5 Google Scholar

Google Scholar Results

Date: 30/05/2023

("new onset" OR newly) AND ("connective tissue diseases" OR rheumatic OR arthritis) AND (associated OR post OR following OR after) AND (covid OR SARS) AND vaccination -medline -embase

Limited to 2019 onwards

~14,100

## A.6 **Manual search**

Date: 09/06/2023
